# Therapeutic Applications of Programmable DNA Nanostructures

**DOI:** 10.3390/mi13020315

**Published:** 2022-02-17

**Authors:** Seaim Lwin Aye, Yusuke Sato

**Affiliations:** Frontier Research Institute for Interdisciplinary Sciences, Tohoku University, Sendai 980-8578, Japan; seaim.lwin.aye.c2@tohoku.ac.jp

**Keywords:** DNA nanostructure, DNA nanorobots, DNA nanotechnology, drug delivery, cellular targeting, cell membrane, smart medicines, cancer therapy, gene therapy

## Abstract

Deoxyribonucleic acid (DNA) nanotechnology, a frontier in biomedical engineering, is an emerging field that has enabled the engineering of molecular-scale DNA materials with applications in biomedicine such as bioimaging, biodetection, and drug delivery over the past decades. The programmability of DNA nanostructures allows the precise engineering of DNA nanocarriers with controllable shapes, sizes, surface chemistries, and functions to deliver therapeutic and functional payloads to target cells with higher efficiency and enhanced specificity. Programmability and control over design also allow the creation of dynamic devices, such as DNA nanorobots, that can react to external stimuli and execute programmed tasks. This review focuses on the current findings and progress in the field, mainly on the employment of DNA nanostructures such as DNA origami nanorobots, DNA nanotubes, DNA tetrahedra, DNA boxes, and DNA nanoflowers in the biomedical field for therapeutic purposes. We will also discuss the fate of DNA nanostructures in living cells, the major obstacles to overcome, that is, the stability of DNA nanostructures in biomedical applications, and the opportunities for DNA nanostructure-based drug delivery in the future.

## 1. Introduction

Advances in nanomedicine have led to the development of advanced therapeutic agents with new therapeutic functions, such as proteins, peptides, monoclonal antibodies, nucleic acids, and live cells. Drugs for precision medicine require solubility, stability, immunity, organ specificity, non-cytotoxicity, easy cellular uptake, and controlled release. Concurrently, new drug delivery strategies are needed to address these challenges by exploiting several technologies and methods, including physical methods, viral vector-mediated delivery [1], and nanoparticle-mediated delivery [2]. Nanocarrier development for drug delivery and therapeutics applies nanoparticles (NPs) in organic forms, such as lipid-based nanoparticles (LNPs) [3,4,5], inorganic forms, such as gold nanoparticles (AuNPs) [6,7,8], polymeric nanoparticles, and polymer-lipid hybrid nanoparticles. A relatively recent strategy is the application of DNA-based nanostructures through DNA nanotechnology as controllable drug carriers and drug delivery systems. What sets DNA nanotechnology apart is that the technique offers nanoscale dimensions, programmability, biocompatibility, and the ability to functionalize DNA. This review focuses on drug delivery carriers and nanorobots for therapeutics based on self-assembled DNA nanostructures (DNSs) in terms of strategy, design, efficiency, and potential. DNSs, which are nanoscale structures made of sequence-designed DNAs, can be customized to program desired sizes and shapes for the desired applications including therapeutic ones.

DNA, the carrier of hereditary genetic information, can be used as a building block for synthesizing nanosized particles of desired shapes and sizes, owing to its unique chemical and structural properties. DNA nanotechnology employs DNA as a non-biological material for the self-assembly of nanoscale structures [9]. The first demonstration of a large-scale structure in 1998 involved the self-assembly of multiple oligonucleotides into nanoscale DNA tiles into higher-order periodic superstructures or DNA lattices of micrometer sizes [10]. Since then, such assemblies have been further constructed to form two- or three-dimensional structures of desired sizes, shapes, and conformations. In 2006, the invention of DNA origami further advanced DNA nanotechnology [11]. The strategy of creating DNA origami involves the application of a long single-stranded scaffold strand that can be folded into the desired structure by binding with many short single-stranded DNA oligonucleotides called staples. The size of the origami depends on the length of the scaffold, which can range from a few hundred nucleotides to several thousand nucleotides. This technique was further adopted and generalized for the self-assembly of the desired DNSs. With an intensive focus on DNA nanotechnology, it is of utmost importance to prove its potential applications with strong merits for practical applications. Aside from the obvious programmability of DNSs for accurate designs, it should also be demonstrated as a functional element in practical engineering devices. Potential applications can be set in programming biochemical pathways using logic gates, the design and implementation of sensing and computing nanodevices, and as tools for delivering therapeutic molecules to target cells with a controlled release.

## 2. Fate of DNA Nanostructures in Living Cells

### 2.1. Targeted Drug Delivery

One of the most important functions of a drug carrier is recognizing and attacking the target cells without interacting with healthy cells while simultaneously traveling through the body and maintaining structural integrity. Therefore, smart nanocarriers, such as nanorobots, must achieve targeted drug delivery or controlled drug release. As in the modification for cellular uptake, targeting a specific cell can be carried out by coupling delivery vehicles with ligands that bind to specific receptors commonly expressed at high levels in diseased cells. Depending on the targeted treatment, these ligands can be aptamers, antibodies, peptides, or other molecules. Aptamers [12], synthetic single-stranded DNA or RNA oligonucleotides, are capable of specific, high-affinity binding to a target that acts as a nucleic acid version of an antibody. Therefore, DNA aptamers can easily function as ligands for DNSs. For example, a DNA aptamer that can recognize a malaria protein biomarker has been combined with a rectangular DNA origami scaffold to serve as a malaria diagnostic material [13]. Peptides have been shown to enhance the efficacy of drugs while reducing toxicity to the cell [14]. Moreover, they can also be used for condensation to efficiently deliver DNA into cells by binding peptides onto the DNA chain through electrostatic interactions and self-associating into β-sheets through hydrophobic interactions and hydrogen bonding [15].

**Figure 1:**
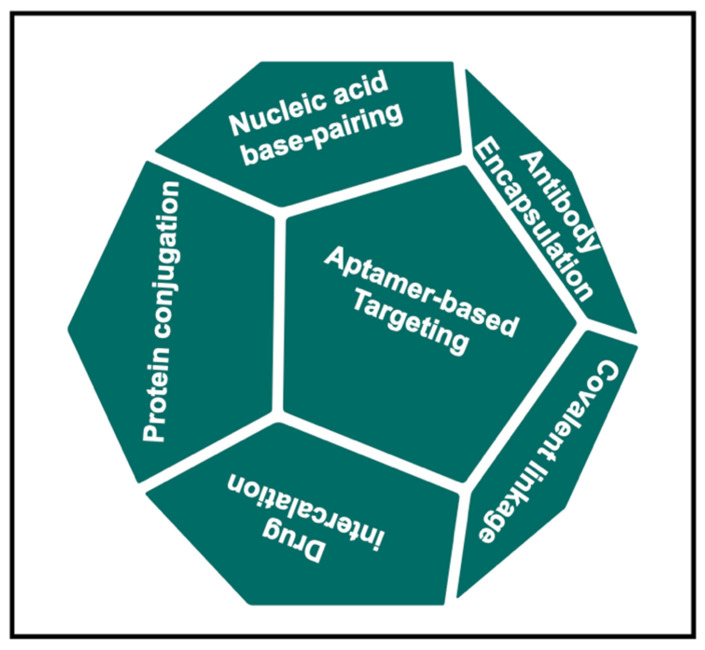
Scheme of self-assembled DNA nanocarriers for drug delivery using various cargo-loading strategies.

In cancer treatment, selective targeting of drugs to tumors is achieved by conjugating a drug to tumor-specific antibodies [16]. One study reported the rational design of a modular DNA-based nanomachine that can load and release its cargo upon binding to a specific antibody by using three different antigens, suggesting the potential application of such a design for controlled drug release [17]. Other receptors such as folate receptors can also be used to target tumors, for example, by coupling to a high-affinity ligand such as folic acid or by coupling to a monoclonal antibody against the receptor of interest [18]. One major advantage of DNS is its suitability for targeted delivery, owing to its highly programmable nature. This can be achieved by programming and determining a suitable size for the DNS by precisely controlling the spatial orientation of the targeting ligands on the structure of DNA. The modification scheme of DNS for therapeutic purposes via various cargo-loading strategies is shown in Figure 1.

### 2.2. Cellular Uptake of DNA Nanostructures

**Figure 2:**
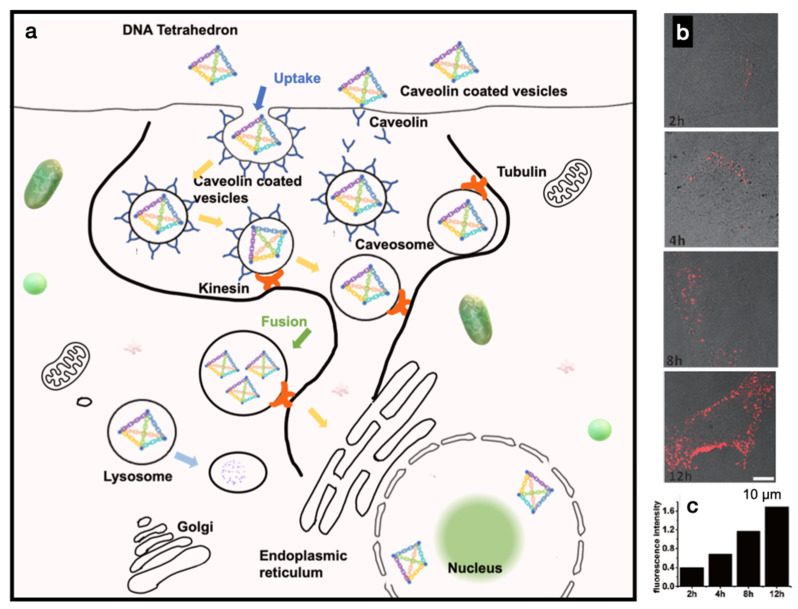
(**a**) Schematics of the cellular uptake, transport, and fate of TDNs. (**b**) Internalization of TDNs by HeLa cells treated with Cy3-conjugated TDNs for 2, 4, 8, and 12 h. (**c**) Flow cytometry analysis of cellular uptake of Cy3-TDNs. Adapted with permission from [24] Copyright © 2022, WILEY-VCH Verlag GmbH & Co. KGA, Weinheim.

To improve the target specificity and cellular uptake of DNSs, structural modifications such as conjugation with a ligand or a transfection agent are usually required for mammalian cell internalization. For instance, DNA nanotubes have been conjugated with folate and Cy3 for cancer cell uptake [19]. With folate conjugation, DNA accumulation was observed at specific cellular locations and perinuclear regions; however, the mechanism of intracellular transportation is unknown. The uptake of DNS by cells can also be achieved through the action of DNA alone. For example, DNA tetrahedron cages have been shown to be taken up by human embryonic kidney (HEK) cells with or without a transfection agent [20]. In this case, the DNA tetrahedra were in the cytoplasm and remained intact within the cells for at least 48 h after transfection. However, the mechanism underlying this substantial uptake remains unknown. Hamblin et al. demonstrated increased cellular uptake by using DNA nanotubes produced via rolling circle amplification with increased uptake of double-stranded DNA into HeLa cells [21]. They suggested that a dense arrangement of a shell of DNA strands in core-shell structures can contribute to cellular uptake without the aid of a transfection agent, which has been similarly carried out before [22]. These findings uncovered the surprising factor that large and highly negatively charged DNSs can enter a cell even without conjugation with transfection agents; however, the mechanism of cellular uptake still remains elusive. Through endocytosis, particles less than 500 nm in size first bind to the plasma membrane and are internalized via receptor-mediated pathways [23]. Liang et al. reported the cellular entry, transport, and fate of tetrahedral DNA nanostructures (TDNs) as receptor-mediated endocytosis specifically mediated by caveolin [24]. They reported that internalized TDNs do not diffuse freely in the cytoplasm but are rather transported through microtubules, indicating that TDNs are transported in an ordered manner through molecular motors (kinesin and dynein) (Figure 2a) [25,26]. Confocal images of HeLa cells treated with TDNs over a period of time of 2–12 h, as well as flow cytometry analysis of cellular uptake, are shown in Figure 2b,c. They also reported that TDNs are eventually trapped within lysosomes, meaning that TDNs are recognized as foreign substances rather than as genetic material.

It will be problematic to employ DNSs for therapeutic purposes if it results in lysosomal degradation. The size of caveolae vesicles ranging from 50 to 60 nm may only carry very small particles [27], limiting the pathway to DNSs of certain sizes. However, to overcome the lysosome degradation issue, Liang et al. further modified TDNs with signaling peptides to direct them to specific organelles such as the nucleus. This indicates that DNSs could be further modified to enhance cellular uptake or to direct them to the desired organelles. For example, coating rectangular DNA origami with virus capsid protein (CP) for transfection into HEK cells improved their delivery into cells by 13-fold compared to bare DNA origami [28]. One study mimicked the morphology of enveloped virus particles for design by encapsulating a DNA nano-octahedron inside a PEGylated lipid bilayer [29]. By enveloping DNSs in lipids, nuclease digestion was eliminated, immune activation was decreased, and bioavailability was increased 17-fold compared with the control. Coating DNSs with proteins such as BSA can also increase transfection into the human embryonic kidney while attenuating the activation of the immune response [30,31]. These findings suggest that coating DNA with DNA-binding proteins could provide a way to increase nanoscale rigidity while protecting it against enzymatic attack and elevated temperatures. DNA provides programmability, and proteins function as nanoscale structural rigidifiers. Moreover, compared to the design of DNSs that cross multiple DNA double helices in parallel to form stiff multi-helical bundles or sheets or using a scaffold for the organization of DNA strands, using hybrid protein–DNA complexes might be a simpler strategy. For a broader topic of building hybrid protein–DNA nanomaterials, we point to a review by Armando Hernadez-Garcia [32].

### 2.3. Stability of DNA Nanostructures in Physiological Conditions

The major challenge in the in vitro and in vivo applications of DNSs is the denaturation of DNSs with the depletion of divalent cations. Moreover, fetal bovine serum (FBS), the cell culture medium used in cells in vitro, contains nucleases that can potentially result in the digestion of DNA. To address this issue, Hahn et al. [33] focused on determining the stability of DNA using in vitro tissue cultures to prototype DNA nanorobots. They tested three different structures: a DNA nano-octahedron (45 nm), a six-helix bundle nanotube (400 nm), and a 24-helix nanorod (16 × 89 nm) with different concentrations of Mg^2+^. They reported that the sensitivity of nanostructures to divalent cation depletion depends on their design and duration of treatment. Among the tested structures, only one (DNA nanotube) was stable and remained intact after 24 h at 37 °C. The addition of MgSO_4_ with an equivalent dilution to prevent osmotic shock provided a viable option for maintaining DNS integrity. While FBS is stored at 4 °C, the level of nuclease activity disappears over days to weeks; hence, it is only a concern for a short period following the preparation medium. In total, 1–2.5% of FBS had little capacity to digest DNA at a 5 nM concentration over 24 h. Therefore, low serum concentration could be used instead of a short incubation time with FBS.

Keum et al. tested the resistance of DNSs to DNase I by comparing digestion patterns between TDNs and linear DNA structures [34]. In their study, TDNs were found to be up to three times more resistant than double-stranded DNA. Different nuclease digestions of DNA origami showed that the degradation rate of DNase I was several hundred-fold slower than that of duplex DNA, suggesting that DNA origami is more stable than smaller TDNs. DNSs can withstand degradation by nucleases better than simple linear DNA, probably because of their interconnected and compact structural design. Mei et al. tested the fate of DNA origami in a rectangular shape (90 nm × 60 nm), a 2D equilateral triangular structure (120 nm × 30 nm), and a 3D multilayer rectangular structure (8-helix × 8-helix square lattice with dimensions of 16 nm × 16 nm × 30 nm) in cell lysates from normal and cancerous cell lines [35]. Their results demonstrated that, in contrast to natural, single- and double-stranded DNA, DNA origami nanostructures (DONs) could be easily separated from lysate mixtures and are fully intact after separation. In this case, the robustness of the DNA origami might be due to the presence of a higher concentration of Mg^2+^ in the assembly process than what might be expected in the cell.

Most of the time, the physiological temperature of 37 °C is used for biological operations; hence, the stability of DNSs at 37 °C for multiple hours should be adequate, depending on the application. According to these findings, the resistance of DNSs to nucleases is related to their structural design. The more compact the structure, the more it can tolerate the attack of nucleases than linear DNA. However, the size of DNSs and their complexity should be considered because a complex design might negatively contribute to cellular uptake and cell biocompatibility. In terms of low or high concentrations of divalent cations, there should be a compromise between the amount required for the structural integrity of DNA and the amount normally present in the body. Several attempts and modifications have been made to overcome nuclease degradation (Table 1). Most experiments used culture media to test in vitro and ex vivo conditions. Such results may not be entirely adaptable in vivo given different physiological conditions in the human body, such as temperature, enzymatic presence, immune response, among others.

## 3. Molecular Payloads

**Figure 3:**
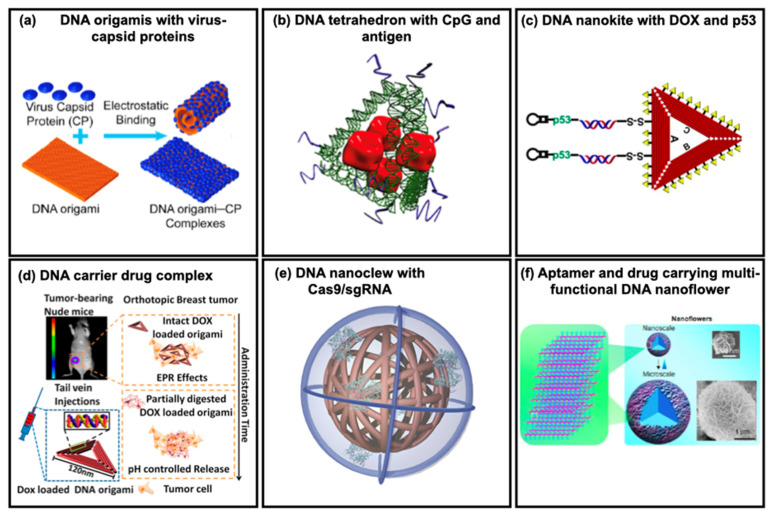
Drug delivery systems based on DNA nanostructures. (**a**) Self-assembly of DNA origami with virus capsid proteins (CPs) to increase transfection efficiency to the cell. Adapted with permission from [28]. Copyright © 2022, American Chemical Society. (**b**) Tetrahedral DNA nanostructure with CpG and antigen as a synthetic vaccine complex. Adapted with permission from [49]. Copyright © 2022, American Chemical Society. (**c**) A DNA nanostructure-based co-delivery system containing a linear tumor therapeutic gene (p53) and a chemotherapeutic drug (doxorubicin, DOX) for the combined therapy of multidrug-resistant tumors (MCF-7R). Adapted with permission from [50]. Copyright © 2022, American Chemical Society. (**d**) DOX/DNA origami complexes injected into the tail of tumor-bearing mice was delivered through blood circulation to accumulate in the breast tumor of mice due to enhanced permeability and retention (EPR) effects. Adapted with permission from [51]. Copyright © 2022, American Chemical Society. (**e**) Yarn-like DNA nanoparticles synthesized via rolling circle amplification for the delivery of a CRISPR system (Cas9/single guide RNA complex). Adapted with permission from [52]. Copyright © 2022, WILEY-VCH Verlag GmbH & Co. KGA, Weinheim. (**f**) Multifunctional DNA NF generated by rolling circle replication can be integrated with aptamer and drug. The diameters of NFs range from ~200 nm to several micrometers. Adapted with permission from [53]. Copyright © 2022, American Chemical Society.

A collection of multiple DNA structures and designs for published drug delivery systems is shown in Figure 3. All the included studies have the same underlying principle and purpose of delivering the payload effectively with enhanced efficiency while protecting it from degradation by external factors during transport. The payload choice depends on the type of targeted disease, the outcome of the therapeutic effect, and the ability of the payload to integrate into the DNSs. The categories of payloads include (i) small molecules such as DOX, (ii) nucleic acids, aptamers and ligands, DNAs and RNAs, (iii) proteins and peptides, and (iv) other molecules such as metals and biotins. Here, we will discuss the payloads that can be categorized as biocompatible and therapeutic agents in more detail. DNSs specifically targeted for application in cancer therapy are listed in Table 2. While most of the studies included are still in the in vitro, ex vivo, or animal model stage, TDN was found to be the most commonly used DNS and DOX as the commonly used drug. Most structures are conjugated with ligands and receptors, such as aptamers and folate, to improve specificity and cellular uptake.

### 3.1. Doxorubicin

DNSs have largely been applied as carrier systems to deliver DOX, a potent anti-cancer drug used to treat a wide range of cancers. DOX can non-covalently bind to double-stranded DNA through intercalation into the helix and is attributed to the convenience of using DNSs for DOX delivery to cancer cells [54]. Jiang et al. independently applied DNA origami structures to deliver DOX into MCF7 cells, a human breast adenocarcinoma cancer cell line [55]. They applied Watson–Crick base pairs in double helices as docking sites for DOX intercalation. Via confocal fluorescence microscopy analyses, the internalization of DOX-origami structures and the co-localization signals from both the drug and carrier were found in the cytoplasm after 24 h of treatment. Both free and origami-coupled drugs effectively induced cell death in a regular cell line. The free drug and drug-loaded dsDNA could not kill drug-resistant MCF7 cells, whereas the origami-bound drug caused cancer cell death, indicating that the carrier-coupled drug can overcome drug resistance. Drugs with DNSs can enhance their cellular uptake, thereby overcoming their decreased cell internalization, leading to the circumvention of drug resistance. Theoretically, the release of loaded drugs can be achieved through the slow degradation of DNSs by low environmental pH or DNA-degrading enzymes, contributing to the potential for controlled drug release.

Similarly, via drug-DNA intercalation, another attempt at DOX delivery into three different breast cancer cell lines was performed using two different DONs of 18-helix bundle nanotubes which are a straight nanotube (10.5 bp per turn) and a twisted nanotube (12 bp per turn) [56]. The structures were designed with varying degrees of global twists to achieve different degrees of relaxation in the DNA double-helix structure. Compared to free DOX, the twisted nanotube structure performed better in encapsulation efficiency and drug release rate, increased cytotoxicity, and decreased intracellular elimination rate. An L-DNA tetrahedron nanostructure (L-TDN), where L is the mirror form of the naturally occurring D-DNA, was used to deliver DOX into cancer cells in vitro and tumor-bearing mice in vivo with previous findings of higher cell penetration than D-TDN [57]. Between L-TDN molecules of two different sizes, one with 17-mer per side and another with 30-mer per side, they found that smaller L-TDNs can enhance drug accumulation in tumors at low doses compared to larger ones.

While DNSs undoubtedly function as DOX carriers, their functionality can be enhanced via modification with an aptamer that can bind to overexpressed molecules on certain cell surfaces to improve specificity and enhance cellular uptake. By modifying TDN-DOX with the aptamer sgc8c, a short DNA sequence that can target protein tyrosine kinase 7, one study delivered DOX to PTK7-positive human T cells CCRF-CEM [58]. They suggested that PTK1-positive CCRF-CEM cells were more cytotoxic than PTK7-negative Ramos cells upon treatment with the sgc8c-TDN:DOX complex. Another use of DNA origami as a DOX carrier was tested with three different origami shapes, a triangle (120 nm), a square (90 nm × 90 nm), and a tube (380 nm). These origami–drug complexes were injected into tumor-bearing mice [51]. Through in vivo and ex vivo imaging, they indicated that DNA origami possesses enhanced tumor targeting and long-lasting accumulation in the tumor region. Among the three structures, the triangle-shaped DNA origami showed optimal accumulation, where the signal mostly remained in the tumor. The square and tube-shaped DNA origami were primarily distributed in the tumor, liver, and kidneys.

Similar to aptamers, folic acid can be used to modify TDN carriers. In one study, DOX intercalation with TDNs was coupled with folic acid to target HT-29 colon cancer cells expressing folate receptors [59]. This strategy increased the cellular uptake of the drug in the presence of folic acid-DNA:DOX compared to that without folic acid, suggesting facilitated penetration through the membrane. The SL2B aptamer, which can inhibit cancer cell growth by disturbing the vascular endothelial growth factor (VEGF) signaling pathways, was used as an additional modification to the TDN to target colorectal cancer [60]. In this design, the TDN was modified with folate and SL2B. Upon encountering the cell, SL2B binds to VEGF165 and inhibits cancer cells growth by interfering with VEGF signaling pathways. Folate–receptor interactions can enhance the cellular uptake and subsequent delivery of DNSs via receptor-mediated endocytosis. Such a combination of nucleic acids and chemotherapy, along with receptor-mediated enhanced cellular uptake, drastically increased the intracellular concentration of DOX over a thousand-fold more than free DOX. Different F and SL2B modifications resulted in varied cell inhibition, where TDN-DOX-2F2S showed significantly higher HT-29 cell inhibition than free DOX, TDN-DOX-2F, or TDN-DOX-FS. TDN-DOX-S also induced more cell death than TDN-S, indicating a synergistic effect between the aptamer and the drug.

Another use of aptamers was reported in a DNA nanocentipede (DNC), where the long trunk was loaded with DOX and the legs were SMMC-7721 cell-binding aptamers (Zy1) that can target cells more firmly and selectively [61]. Flow cytometric analyses demonstrated that Zy1 with DNC was more effective in terms of binding affinity and selectivity than free Zy-1. Multidrug resistance (MDR) protects a tumor cell against several drugs with different chemical structures and mechanisms of action [62]. Mei et al. applied a DNA nanoflower (DNF) with a tunable size of up to 200 nm in diameter to deliver DOX to MDR cancer cells and chemosensitive cells [63]. NFs can self-assemble via the liquid crystallization of DNA generated through rolling circle replication, during which aptamers, fluorophores, and DOX are incorporated. DOX-loaded NFs were found to be stable at physiological pH, and drug release was facilitated by either acidic or basic conditions. They reported that NFs delivered DOX into chemosensitive and MDR cells, inducing potent cytotoxicity, while non-target cells were left unharmed. Kim et al. previously demonstrated the targeting of MDR using a DNA tetrahedron for the delivery of DOX into drug-resistant breast cancer cells [64]. Interestingly, Liu et al. combined chemotherapy with gene therapy by co-delivering DOX with a linear tumor therapeutic gene (p53) and a DNA origami targeting a multidrug-resistant tumor (MCF-7R) [50]. The design resembles a kite (a nanokite) where DOX is intercalated within the triangular space of a triangular origami with a protruding disulfide linker hybridized with p53. The images of excised tumors from mice after 24 days of treatment showed a drastic decrease in the size of tumors treated with DOX and p53 compared to DOX without the p53 sequence and vice versa. Their findings suggest that such a coupled therapy can not only overcome drug resistance but also demonstrate the potential of DNS as a carrier for gene therapeutics.

**Figure 4:**
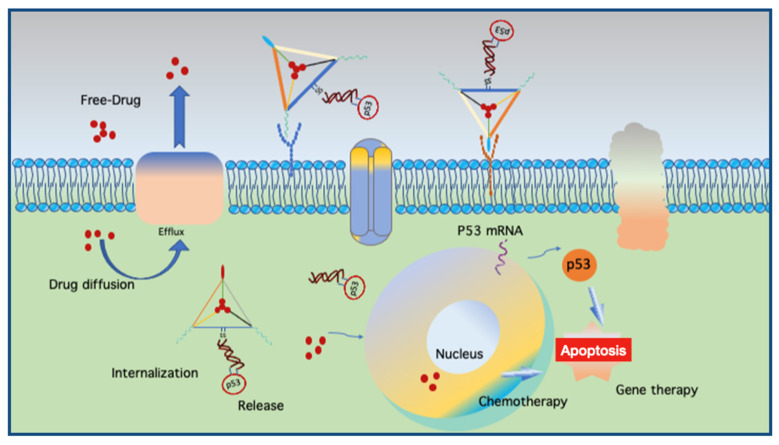
The molecular mechanism involved in circumventing multiple-drug resistant (MDR) cells and the dual therapy of cancer cells. MDR cells excrete drugs via an efflux pump and the DNA nanostructure can deliver the drug into the cell via cellular uptake through endocytosis. The drug is released through pH-dependent conditions and subsequently delivered to the nucleus to induce apoptosis. DNA nanostructures equipped with both drugs and tumor therapeutic genes can co-deliver dual chemotherapeutic and gene therapeutic effects to MDR cancer cells. Illustration inspired from [50].

The above-mentioned findings support the application of DNSs for enhanced drug internalization and the circumvention of drug resistance using a relatively convenient strategy such as click chemistry. By combining chemotherapy and gene therapy (Figure 4), the potential of DNS-based smart therapeutics is increasing as more modifications with ligands for target specificity and enhanced uptake are discovered, while maintaining carrier biocompatibility. However, each approach has a different loading strategy, pH, working environment, and DOX concentration, making it difficult to interpret and compare the results of such findings. Moreover, high concentrations of DOX during the loading process can also lead to the self-aggregation of DOX. Another important factor is the hybridization ability of DOX with self-hybridized staples if excess staples are not eliminated after the folding process. In addition to DOX, another intercalating drug, daunorubicin, a chemotherapeutic agent used to treat leukemia, can be loaded into DNSs. Halley and colleagues employed rod-like horse DNSs to circumvent daunorubicin drug resistance in the leukemia cell line HL-60/ADR with enhanced drug efficacy [65]. They hypothesized that the free drug delivered in solution enters cells via passive diffusion and that the horse nanostructures enter cells via endocytosis. This process leads to larger amounts of the drug in the cell, enhancing drug efficiency while maintaining a clinically relevant concentration of daunorubicin (0.1–1.0 × 10^−6^ M).

**Table 2:** DNA nanostructures in cancer therapy.

Structure	Payload	Modification	Results	Ref.
TDN	DOX	Folate receptor	6 h incubation induced apoptosis of HT 29 colon cancer cells.	[59]
TDN		HApt	Enhanced stability and prolonged circulation of HApt, induced apoptosis and arrested cell growth.	[66]
TDN	DOX	Affibody	Bind ~ 53 molecules of DOX with greater selectivity and inhibition of breast cancer cells.	[67]
TDN	DOX	Folate receptor	A synergic anti-cancer biological effect with chemotherapy.	[59]
TDN	5-FU	AS1411 aptamer	Better targeting ability to kill breast cancer.	[68]
TDN	DOX	AS1411 + MUC1 aptamer	Lower cytotoxicity to MUC1-negative cells, equal lethality to sensitive cells, and more effective to DOX resistant cells.	[69]
TDN	TMZ	AS1411 + GS24	Attenuate drug resistance in temozolomide (TMZ)-resistant cells with specific binding to transferrin receptor.	[70]
TDN	Ir	AS1411 + MUC1 aptamer	Inhibits the growth and invasion of glioma cells.	[71]
TDN	ASOs	Nuclear localization peptide	Antisense strands released inhibit cell proliferation at a low concentration without transfection reagent with efficient knockdown of the *c-raf* gene.	[72]
TDN	DOX		Efficient delivery of DOX into drug-resistant breast cancer cells.	[64]
TDN	DOX	KLA peptide	3KLA-modified TDNs designed for mitochondrial targeting exhibited the most efficient DOX accumulation in mitochondria of 4T1 cells leading to an effective release of cytochrome c, and the upregulated expression levels of caspase-9, caspase-3, p21, and p53.	[73]
NF	DOX		Circumvent drug-resistant cells with less side effects to non-target cells.	[63]
NF	DOX	Sgc8	Preparation of multifunctional DNA Nanoflowers that are resistant to nuclease and can integrate with different functional moieties.	[74]
DNA triangle	DOX		Exhibited remarkable anti-tumor efficacy without systemic toxicity in mice with orthotopic breast tumors.	[51]
DNA triangle	BMEPC		Cellular-level dual-functional imaging and photodynamic therapy that generates free radicals and subsequent apoptosis.	[75]
DNA triangle and tube	DOX		Increased cellular internalization of DOX with enhanced cell-killing activity to drug-resistant adenocarcinoma cells.	[55]
DNA tube with conformational change to DNA sheet	Thrombin	AS1411 aptamer	Nucleolin-targeting aptamer serves both as a targeting domain and as a molecular trigger for the mechanical opening of DNA nanorobot delivering thrombin, specifically tumor-associated blood vessels, and inducing intravascular thrombosis resulting in tumor necrosis and inhibition of tumor growth.	[76]
DNA icosahedron	DOX	MUC1 aptamer	DOX@Apt-DNA-icosa shows efficient and specific internalization for killing epithelial cancer cells.	[77]
DNA dendrimer	EPI	AS1411+ MUC1 aptamer	Apts-Dendrimer-Epi complex released Epi in a pH-sensitive manner (more release at pH 5.5), prohibiting tumor growth in vitro and in vivo.	[78]
DNA nanorod	Daunorub-icin		Circumvent efflux pump-mediated drug resistance in leukemia cells at clinically relevant drug concentrations.	[65]
DNA nanocircuit	Chlorin e6	Aptamer	Aptamer-based DNA nanocircuit selectively recognizes target cancer cells, activates photosensitizers, and amplifies the photodynamic therapeutic effect.	[79]
DNA nanotrain	DOX	AS1411, Sgc8	Locomotives guiding nanotrains with boxcars carrying high payload allowing intracellular signaling.	[80]
DNA nanocentipede	DOX	Zy1	Effective binding affinity and selectivity with enhanced cellular cytotoxicity to the target cell but not to negative control cells.	[61]
X-Y-Shaped DNA	DOX	Sgc8	Specific cytotoxic effect against leukemia cells with the incorporation of therapeutic antisense oligonucleotides inhibiting efflux pump of drug circumventing drug resistance.	[81]
Biotinylated octahedral DNA nanocages	DOX	Folic acid	DOX-loaded Bio-Fol-DNA nanocages delivered DOX selectively to the folate receptor-enriched Hela cells.	[82]
A 3D tubular DNA origami with six helical bundles	DOX	DUPA (a small molecule ligand)	Ligand conjugate DONs delivered DOX with high affinity and selectivity into the prostate-specific membrane antigen (PSMA)+ cancer cell line LNCaP. DOX-DUPA-DONs showed lower toxicity against PC-3 cells (PSMA-) in comparison to free DOX.	[83]
tFNA (Tetral framework nucleic acid)	Maytansin-e (DM1)	HApt-aptamer	HApt-tFNA@DM1 (HApDC) could target HER2 protein and delivered chemotherapeutic agents into HER2-positive breast tumor. HApDCs exerted enhanced anti-tumor efficiency in comparison with free drug and synthetic liposome-derived vesicles without side effects.	[84]
All-sealed divalent aptamer Tetrahedral DNA framework (asdTDF)	Therapeutic protein	Aptamer	The ligase-assisted seal of the nicks resulted in the enhanced TDF stability against nuclease digestion protecting the therapeutic protein from degradation. Endogenous gluathione can trigger the release of therapeutic protein leading to the apoptosis of the specific cancer cells.	[85]
Tetrahedral DNA	Photother-anostic molecule (IR780)		The in vitro and ex vivo photothermal and photodynamic efficiencies of IR780 in the tumor site was high in IR780@Td with enhanced tumor imaging and anti-tumor effects than IR780 alone.	[86]
A triplex-DNA nanoswitch	Drug combo (Antisense DNA, Cisplatin, DOX	Aptamer	The effects of gene silencing and significant inhibition of tumor growth was shown with tumor-bearing mouse models upon intravenous administration of smart pH responsive nanoswitch that can be used for combinatorial cancer therapy.	[87]

### 3.2. Therapeutic Nucleic Acid Delivery

In addition to drug delivery, DNSs have also been employed to deliver functional therapeutic nucleic acids such as aptamers, antisense RNAs, small interfering RNAs (siRNAs), microRNAs, and antisense oligonucleotides.

Cytosine-phosphorothioate-guanine oligodeoxynucleotides (ODNs), which contain phosphodiester links between C and G nucleotides, are potent activators of innate and acquired immune responses. CpG sequences that are more abundant in bacterial genomes than in mammalian genomes [88] are considered pathogenic signals by the immune system and can stimulate Toll-like receptor 9 (TLR 9), resulting in the secretion of inflammatory cytokines [89], leading to immunotherapeutic properties. Due to their susceptibility to nucleases, CpG sequences alone cannot reach the desired target sites. Therefore, modifications have been designed to achieve stability, such as phosphorothioate (PS) backbones, high-order tertiary structures via the formation of poly(G) motifs, and PS backbones in dumbbell-like structures [90]. Nishikawa et al. prepared Y-shaped oligodeoxynucleotides (Y-ODNs) using three ODNs with half of each ODN partially complementary to half of the other two ODNs [91]. Y-ODNs induced a higher level of tumor necrosis factor-α and interleukin-6 from RAW264.7 macrophage-like cells and higher cytokine levels than dsODNs containing identical sequences. This Y-shaped DNA was further developed into a larger dendrimer-like structure (DL-DNA) [92,93]. DNA immunonanoflowers (NFs) as multivalent CpG nanoagents were developed from long DNA molecules integrated with tandem CpG sequences through rolling circle replication [94] for efficient CpG delivery and protection from nuclease degradation [95]. Zhu et al. also integrated NFs with aptamers, bioimaging agents, and drug-loading sites as proof-of-principle demonstrations [53]. Mohri et al. assembled multiple CpG sites to form a multi-branch Y-X or dendrimer-like polypod structure [96]. An increasing number of pods (from three to eight pods) is directly linked to better stability, efficient cellular uptake, and increased cytokine production.

**Figure 5:**
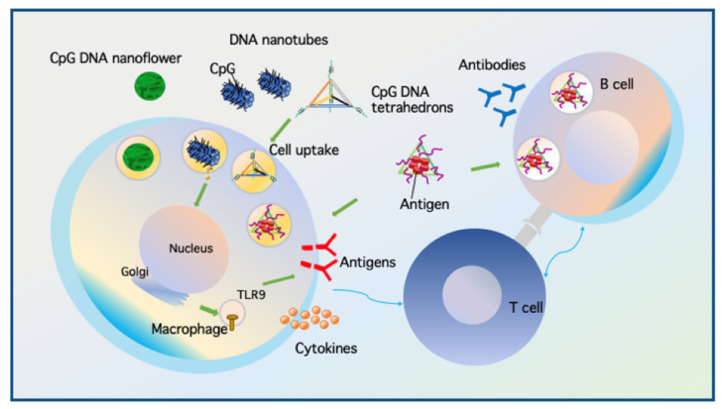
DNA nanostructures act as a synthetic vaccine by carrying CpG ODN with or without antigens to activate immune cells. DNS without antigens enter macrophages and deliver CpG ODN recognized by Toll-like receptor (TLR)-9 that can stimulate the cell to produce antigens and cytokines. DNS with antigens specifically enters B cells and non-specifically to macrophages. T cells then activate the B cell response leading to antibody production. Illustration inspired from [49].

In addition to integrating CpG sequences into a larger DNA sequence, Li et al. employed functional 3D DNA tetrahedra with CpG appendices at each corner to achieve the non-toxic and stable delivery of CpG to RAW264.7 cells [97]. Such a structure can protect CpG sequences from nuclease degradation and remain intact for at least seven hours. After cellular uptake, CpG motifs activate downstream pathways to induce immune responses. Interestingly, Liu et al. applied DNS along with antigen and CpG adjuvants to develop a synthetic vaccine [49]. As in a previous study, CpG sequences were conjugated to the corners of a DNA tetrahedron, and a model antigen (streptavidin) was embedded inside the DNA tetrahedron. From their results, the antigen-CpG-DNS complexes induced long-lasting and robust antibody responses against the antigen without stimulating a reaction to the DNS itself, indicating the potential application of DNSs in developing more effective vaccines. Schuller et al. applied another form of DNS with CpG to investigate the potential of DNA origami constructs as programmable and noncytotoxic immunostimulants [98]. In this study, a hollow 30-helix DNA origami tube (80 nm × 20 nm) was covered with up to 62 CpG sequences and tested for immune responses in freshly isolated spleen cells. Such decorated origami tubes triggered higher immunostimulation than an equal amount of CpG using Lipofectamine, a common transfection agent. They also found a lack of immune response to nanotubes without CpGs and showed no detectable toxicity compared to Lipofectamine. These findings indicate that DNS is a suitable candidate for transporting CpGs into target cells, providing safe and enhanced cellular uptake with less toxicity, thus serving as a better alternative to commonly used transfection agents. DNA can be used to deliver CpG ODNs for immunization purposes with or without antigens (Figure 5).

The AS1411 aptamer, a potential cancer therapeutic agent by itself or in combination with other drugs, was incorporated into DNA pyramids to achieve enhanced cellular uptake and selectivity [99]. DNA pyramids also protected single-stranded aptamers from nucleases while inhibiting HeLa cell growth within 24 h. In cancerous cells, the aptamer alone could enter through the micropinocytosis pathway and escape endolysosomal degradation. In contrast, in non-cancerous cells, AS1411 can end up in the lysosomes. AS1411-pyramids behave similarly to the aptamer alone cellular uptake mechanism of ending up in the lysosomes of normal cells, thereby preventing adverse effects on normal cells.

Lee et al. used TDNs to deliver siRNAs into tumor cells and silence target genes in tumors [100]. By applying folic acid as ligands, they observed that at least three folate molecules per nanocarrier were required for the optimal delivery of siRNAs into cells. Moreover, gene silencing only occurs when the ligands are in an appropriate spatial orientation. Kim et al. applied wireframe TDN with a 20-mer duplex on each side to deliver siRNA into the liver targeting ApoB1 mRNA which is overexpressed in hypercholesterolemia [101]. In vivo and ex vivo images of BALB/c mice showed that duplex siRNA (siApoB1) was able to reach the liver with lower accumulation level than that of Td-siApoB1. Accumulation of Td-siApoB1 in the liver can result in the downregulation of ApoB1 protein leading to the decreased blood cholesterol level. Xue et al. also employed TDNs as building blocks to construct a DNA-based nanogel in which siRNAs and DNA tetrahedra are crosslinked by a specific sticky end to deliver siRNA [102]. A framework DNA tetrahedron with a tail and a single-stranded DNA molecule complementary to each end of the siRNA linkers acts as a building block to mix with siRNA linkers at an optimized ratio of 1:1.8 to assemble into a crosslinked nanosized hydrogel. They stated that the nanosized 3D nanogel prevents the nuclease digestion of the loaded siRNA; however, at the same time, RNase H-mediated cleavage can release the siRNA inside the cell. Similarly, Fu et al. developed a smart pH-responsive DNA nanohydrogel system as a carrier for the delivery of mRNA into HeLa cells [103]. They designed X-shaped DNA scaffolds and DNA linkers with i-motif sequences to crosslink the target mRNA to form the nanohydrogel with a compact spherical shape. The dehybridization with the scaffolds occur at an acidic pH (pH 4.5–5.0) releasing the mRNA. They claimed that the nanohydrogel system showed better biocompatibility and comparable mRNA expression efficiency relative to the commercial liposome. Such a system can become an alternative to the liposome for delivering small RNA molecules.

RNA interference (RNAi) is a therapeutic strategy that induces gene silencing by targeting disease-causing mRNAs removed through degradation pathways. Fakhoury et al. applied 3D DNA cages in the shape of a triangular prism (TP) integrated with nucleic acid therapeutics, an antisense oligonucleotide for firefly luciferase, at one, two, four, or six sites for transfection into HeLa-Firefly Luciferase cells [104]. The outcome was superior to that of single-stranded and double-stranded controls, with a slight premature dissociation of the antisense oligonucleotides from the DNA scaffold. TPs with four and six antisense strand positions maintained gene silencing up to 72 h and were more robust at gene knockdown after removal. For the encapsulation and conditional release of siRNA, Bujold et al. designed DNA nanosuitcases that can enclose a siRNA construct and release it upon recognizing mRNA or microRNA (miRNA) oligonucleotide RNA [105]. Upon recognizing the marker, the two gating strands were unwound via strand displacement, releasing the siRNA [106]. The design can be modified for dual therapy purposes, with the gating strands as antisense strands performing gate opening and gene silencing. Such a design was reported to be effective in increasing half-life, protecting siRNA, controlling release, and having the potential for diverse applications with logic gates that can be tailored to the biological system of interest.

### 3.3. Delivery of Gene Editing Tools

**Figure 6:**
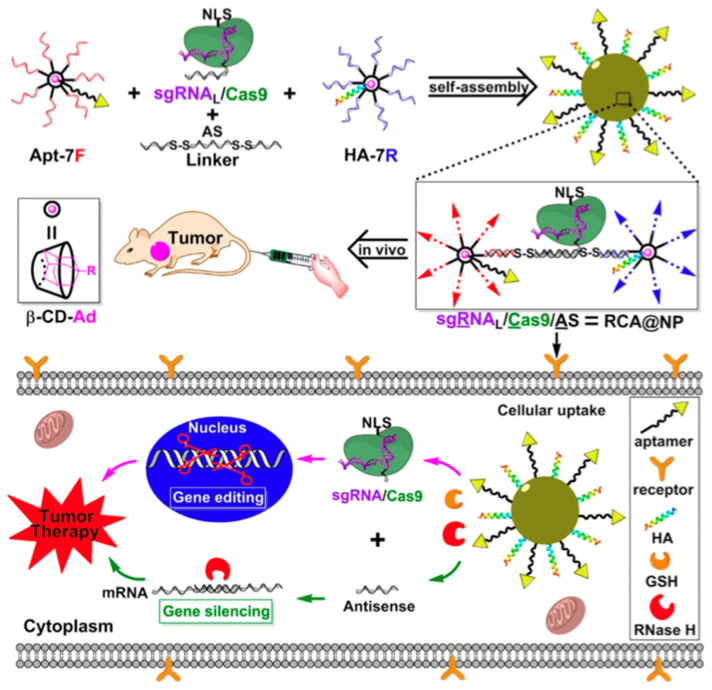
Model of DNA nanostructure that co-deliver gene editing and gene silencing to the cell. DNA nanostructures base-paired with the sgRNA/Cas9 and covalently crosslinked with antisense strands enter the cell via endocytosis. Antisense strands are released by RNase H and bind to messenger RNA for gene silencing. The CRISPR complex enters the nucleus to perform gene editing while the multifunctional nanocarrier provides synergistic tumor therapy. 7F or 7R: DNA oligonucleotides covalently crosslinked by beta-CD. Reproduced with permission from [108] Copyright © 2022 American Chemical Society.

The RNA-guided Cas9 nuclease from the microbial clustered regularly interspaced short palindromic repeat (CRISPR) immune system can facilitate gene editing and genome engineering in eukaryotic cells by simply specifying a 20-nucleotide targeting sequence within its guide RNA [107]. The CRISPR/Cas genome editing system can be engineered to target almost any gene of interest with precise and efficient gene editing in various cells. The major obstacle in its application is the delivery of the system to target cells. Currently, viral vectors are the most used vehicles for cell delivery, but they can also contribute to the adverse effects of genetic diseases and off-target side effects. Sun et al. synthesized DNA nanocarriers via rolling circle amplification to transport a Cas9/sgRNA complex into the cell nucleus [52]. Yarn-like nanocarriers were loaded with Cas9/sgRNA complexes through Watson–Crick base pairing. This, in turn, was encapsulated in a coating of the cationic polymer polyethyleneimine to help induce endosomal escape. Nuclear transportation was achieved via nuclear-localization-signal peptides fused to Cas9. According to the flow cytometry results, the mutation frequency in cells treated with DNA nanocarriers was 18-fold higher than that in cells without DNA nanocarriers. They reported that the partial complementarity between DNA nanoclews and sgRNA guide sequences promoted the extent of gene editing probability by balancing the binding and release of the Cas9/sgRNA complex. Liu et al. employed a branched DNA nanoplatform via covalent crosslinking to deliver the sgRNA/Cas9/antisense complex for synergistic gene silencing and tumor therapy in vitro and in vivo [108]. They also incorporated aptamers for cell targeting and a peptide for endosomal escape, attempting to achieve the anti-tumor effects of gene editing (DNA in the nucleus) and gene silencing (mRNA in the cytoplasm) in vivo (Figure 6). These findings indicate the potential of DNA nanotechnology in genome editing in the future, and similar purposes of CRISPR/Cas9 delivery can be achieved using virus-like designs of DNA nanocarriers.

## 4. DNA Nanorobots That Deliver Molecular Payloads

**Figure 7:**
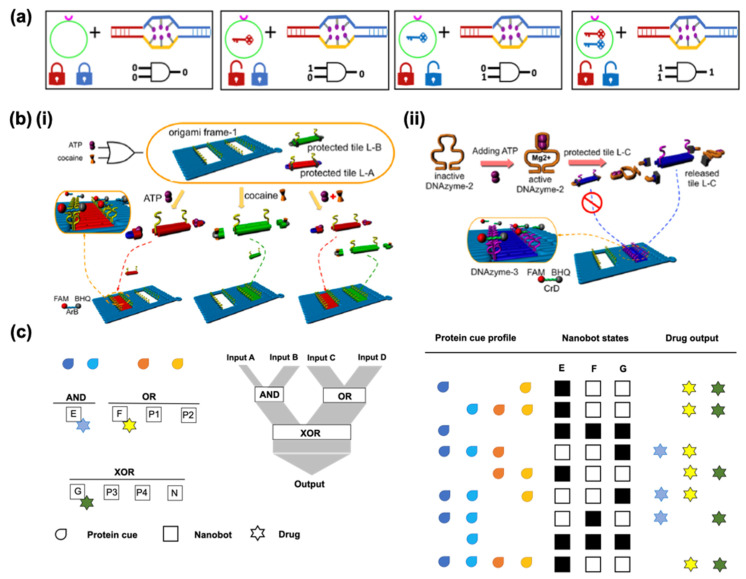
Logic-gated nanorobots; inputs (A, B), outputs (1,2). (**a**) Nanorobot activation via an AND logic gate. The aptamer-encoded locks respond to molecular input (key)-expressing cells leading to the conformational change of the nanorobot as an output. Redrawn from [109]. (**b**) Aptamer-binding directed DNA origami pattern for logic gates: (**i**) operation of an OR logic gate through a DNA origami using ATP and cocaine as two independent inputs to trigger the filling patterns, and (**ii**) operation of a two-layer YES gate where an active DNAzyme is designed to leave the protected tile to prevent direct filling into the origami. Adapted with permission from [114]. Copyright © 2022, American Chemical Society. (**c**) A hypothetical system consisting of eight robot types capable of simultaneously controlling three therapeutic molecules: three effector robots E, F, and G, each carrying a different drug; four positive regulators, P1 and P2 keying F, and P3 and P4 keying G; and a negative regulator N inactivating G forming two first-layer gates, AND and OR, each controlling a respective drug while relaying its outputs to a second-layer XOR gate that controls the third drug. Redrawn from [113].

The controlled release of drugs has been achieved with diverse nanomaterials that can react to environmental stimuli such as variations in pH, temperature, and magnetic field strengths. Using several approaches, such materials can not only respond to biochemical or physical stimuli but can also be programmed to use logic gates for analysis. In biocomputing based on the interactions of biomolecules, different approaches can be applied to create logic gates, identify general algorithms, and obtain output signals from the inputs. The implementation of logic-gated systems in DNS was pioneered a decade ago by Douglas et al. who designed a prototype of DNA origami-based nanorobots for the smart delivery of molecular payloads [109]. They adapted a DNA box with a controllable lid from a previous report [110] to use as a 3D DNA box in the form of a hexagonal barrel with dimensions of 45 × 35 × 35 nm^3^. The barrel consists of two domains, in the form of an empty box and a lid, where single-stranded scaffold hinges are located at the back and staples modified with DNA aptamer-based locks are located at the front. In this case, (Figure 7a), the aptamer is a lock that can be opened by binding to the antigen keys, which are designed to operate in response to proteins, based on [111].

Structure-switching aptamers undergo target-induced switching between an aptamer–complement duplex and aptamer–target complex. When aptamers recognize their targets, the lock duplexes dissociate and the nanorobot undergoes a drastic reconfiguration. Molecular payloads can be loaded through a short ssDNA oligonucleotide linker inside the nanorobot. They are intended to load at least two molecules per robot for multivalent interactions with the surface receptors [112]. Dogulas et al. applied gold nanoparticles and Fab antibody fragments that can attach to a protein marker on the surface of the cell of interest so they can consequently interact. These agents were programmed to be activated in response to a single input using the same aptamer sequence on the right and left sides. If different aptamers are used, the nanorobot can only be opened in the presence of two inputs; in other words, two different keys are required to open the two locks. These logic gates of inputs of binding or non-binding (0 or 1) that lead to outputs of closed or open states (0 or 1) in nanorobots are equivalent to a logical AND gate. Amir et al. designed a system with various logic gates, such as AND, OR, and XOR, with DNA origami robots in living cockroaches to control molecules that target their cells [113]. Hypothetically, their system serves as a processor that can deliver a therapeutic response to a different disease state based on a selection of three drugs. The system consists of eight robot types: three effector robots, each carrying a different drug, four positive regulators and a negative regulator. They altogether set up two first-layer gates, AND and OR, each controlling its own drug relaying its output state to a second-layer XOR gate which controls a third drug (Figure 7c). Four drug combination outputs could be generated by using such a model system. Yang et al. performed a set of logic gates (OR, YES, and AND) in response to the stimuli of adenosine triphosphate (ATP) and cocaine with an aptamer-binding DNA origami pattern [114]. Small DNA tiles were controlled to fill the predesigned DNA origami frame by combining DNA aptamer–substrate binding and DNAzyme-cutting (Figure 7b).

**Figure 8:**
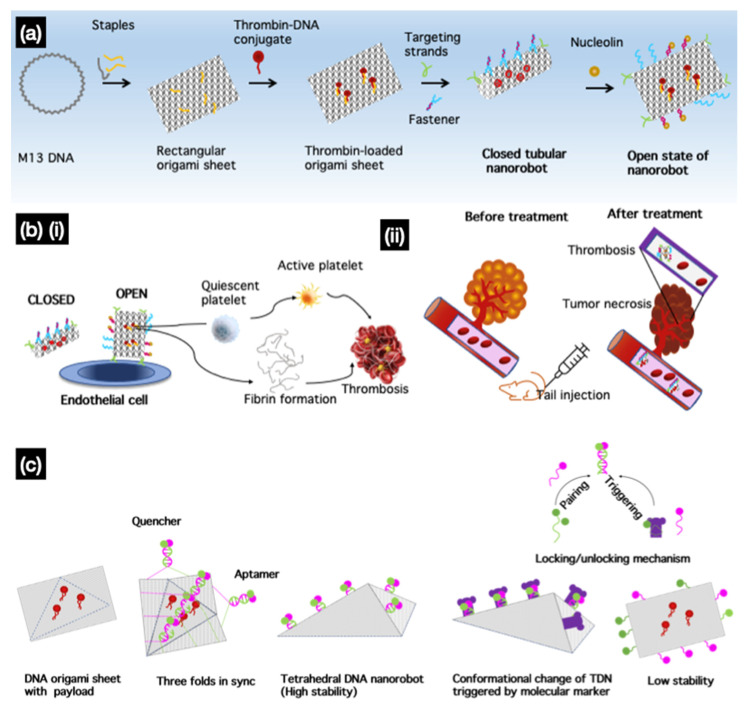
Application of DNA nanorobots for targeted therapy. (**a**) Construction of a nanorobot-Th through DNA origami. The closed tubular nanorobot opens upon sensing nucleolin to open the DNA origami sheet. (**b**) (**i**) The mechanism of action of nanorobot-Th in plasma in the presence of vascular endothelial cells. (**ii**) The therapeutic mechanism of nanorobot-Th within tumor vessels. DNA nanorobot-Th administered to tumor xenografted mice via tail vein injection binds to the vascular endothelium by recognizing nucleolin and opens to expose its thrombin cargo which induces localized thromboses, tumor infarction, and cell necrosis. Redrawn from [76]. (**c**) Dynamic DNA nanostructures that respond to external stimuli can perform a conformational change; a DNA rectangular sheet that can fold synchronously into a tetrahedral DNA nanorobot driven by five aptamer duplexes. Through a locking and unlocking mechanism, in response to epithelial cell adhesion molecule (EpCAM), a TDN undergoes a conformational change back to the DNA origami sheet that exposes its payload (a red fluorescence dye in this case). Redrawn from [115].

One of the most promising applications of DNA nanorobots was reported by Li et al., who applied nanoscale DNA robots as intelligent drug delivery systems that respond to molecular triggers in vivo for cancer therapy. They used a DNA nanorobot that delivered thrombin, a coagulation factor, and a serine protease specifically in tumors [76]. In their DNA nanorobot design, a rectangular origami sheet was prepared from M13mp18 single-stranded DNA with predesigned staple strands. Thrombin was loaded onto the surface of the DNA sheet structure via poly-T and poly-A oligonucleotide hybridization (Figure 8a). When fasteners and aptamers are added, the sheet forms tubular DNA nanorobots that carry thrombin that can target aptamers at both ends. The nanocarrier tube opens when nucleolin, a protein highly expressed in the tumor, is present. In the open state, encapsulated thrombin induced localized thromboses, tumor infarction, and cell necrosis (Figure 8b). They demonstrated that nanorobots not only affected the primary tumor, but also prevented metastasis in a melanoma mouse model. They reported the safety and inert immunological activity of nanorobots in mice and Bama miniature pigs. They also intended to further develop the current strategy as a drug delivery method for treating other diseases by modifying the geometry of the nanostructures, targeting groups, and loaded cargo. Similar to a previous study [76], Liu et al. [115] recently designed a tetrahedral DNA nanorobot that responds to molecular triggers to perform a conformational change when targeting tumor cells. A 2D DNA origami sheet (DOS) was folded into a 3D tetrahedral DNS using multiple parallel-folding elements. The folding of the DOS was aided by five pairs of DNA molecules containing SYL3C aptamer sequences that target epithelial cell adhesion molecule (EpCAM), a molecule specifically expressed on circulating tumor cells. When the aptamers bind to EpCAM, the TDN is triggered by EpCAM to unfold into a DOS to expose the molecules or drugs inside; in this case, a fluorescent dye was used for visualization (Figure 8c).

## 5. Tetrahedral Framework Nucleic Acids as Therapeutic Agents

Recently, tetrahedral framework nucleic acids (tFNA) have been widely used as therapeutic targets for neurological disorders. For example, Li et al. employed aptamer-conjugated framework nucleic acids to repair cerebral ischemia-reperfusion injury (IRI) in an animal model [116]. Oxidative stress and excessive inflammation are the main causes of IRI and can lead to neuronal damage and disability. Complement component 5a (C5a) exacerbates stress and immune responses. By applying a framework nucleic acid (FNA) conjugated with anti-C5a aptamers, which can selectively reduce C5a-mediated neurotoxicity, the group performed an intrathecal injection in mice to reduce oxidative stress. The structure of the framework is equivalent to two pyramids (tetrahedra) stacked on top of each other, with protruding aptamers. They hypothesized that C5a-FNA could function to scavenge free radicals and block C5a-mediated neurotoxicity to inhibit cerebral IRI and found that such conjugates functioned as antioxidants at the cellular level to protect primary neurons from oxidative stress. This study indicated the potential of DNS in neural therapy for several purposes, with suitable modifications for the designated diseases to be cured.

Another example of the application of DNSs in neural science was performed very recently by Zhou et al. in glioma cells using a DNA tetrahedron as a surviving siRNA carrier to combat brain tumors [117]. Another group also constructed a tetrahedral DNS-loaded surviving interfering RNA (As-TDN-R) to selectively identify tumor cells overexpressing nucleolin, which is highly expressed in various tumors and can promote tumor progression [118]. Nucleolin also acts as a ligand for the aptamer AS1411 and supports its cellular entry [119]. Owing to the potential of nucleolin as a glioma marker, since there is a differential expression of nucleolin between glioma cells and normal cells, the AS1411-attached nanostructures showed differences in intercellular uptake, although its exact mechanism remains unclear. The structure equipped with aptamers for cell targeting increased siRNA delivery and efficiently induced apoptosis in glioma cells, which were activated by inhibiting survivin expression. Shi et al. performed similar aptamer-modified tFNA for targeted glioma therapy [120]. They employed tFNA to deliver two aptamers, GMT8 and Gint4.T, and an anti-tumor drug, paclitaxel, into U87MG and bEnd.3 cells without the aid of transfection agents. The linkage of tFNA with aptamers alone and aptamers with the drug showed anti-glioma efficacy. In addition to neurological disorders, Xie and colleagues tested TDNs loaded with paclitaxel to treat drug-resistant lung cancer, where paclitaxel solutions of different concentrations were incubated with TDNs at room temperature for 24 h to recover the drug-loaded white precipitate after centrifugation [121].

Furthermore, tFNAs have been reported to be neuroprotective [122], antioxidant [123], and anti-inflammatory agents [124]. Chen et al. applied tFNA without the conjugation of therapeutic agents or functional agents to target Alzheimer’s disease [125]. While the therapeutic properties of tFNA are not yet fully understood, they reported the inhibition of apoptosis and reduction in amyloid beta proteins in the brain, in addition to the ability to partially pass the blood–brain barrier. They proposed that the function of tFNA in Alzheimer’s disease models involves inhibiting the mitochondria-dependent apoptotic pathway. First, tFNA reduces the level of reactive oxygen species (ROS), thereby reducing the activation of caspases, inhibiting the apoptosis-related signaling pathway, and finally inhibiting apoptosis. Similarly, to explore potential alternative therapies for multiple sclerosis, Yang et al. characterized the effects of tFNA on remyelination [126]. They reported that these nucleic acids could accelerate remyelination and enrich myelinated axons by restoring the expression of myelin-related proteins. Inhibiting apoptosis, in addition to inhibiting the abnormal activation and proliferation of microglia and astrocytes, relieves inflammatory reactions in vivo. Such outcomes were proposed to be obtained via tFNAs upregulating the phosphorylation of the PI3K-AKT-mTOR signaling pathway.

The role of this pathway was also reported by Yao et al. who employed tFNAs to facilitate the restoration of facial nerves [124]. They reported that tFNAs can regulate the neurorestorative pathway in activating a series of cell behaviors related to injured nerve restorations, along with enhanced expression of axon and myelin marker proteins, histological recovery, and muscle movement in vitro and in vivo. Li et al. explored the effect of tFNA on the wound healing using corneal epithelial wound as an example [127]. They reported the enhanced proliferation of human corneal epithelial cells upon exposure to tFNAs in vitro. In vivo experiment with animal model of corneal alkali burns through clinical evaluations and histological analyses showed the improved corneal transparency and re-epithelialization of wounds. The application of DNSs as therapeutics is not limited to tFNAs, other DONs were also shown to apply as such application. Jiang et al. applied radiolabeled DONs with three different shapes, rectangular, triangular, and tubular, as therapeutic agents to treat acute kidney injury (AKI) [128] which frequently requires kidney transplantation. When applied to murine models of induced AKI, DNA origami scavenges ROS, alleviates oxidative stress, protects the kidney, and alleviates AKI. Among the biodistribution patterns of the three different DNA origami, all three performed better than short ssDNA, M13 ssDNA, and partially folded DNA origami. Rectangular DNA origami showed renoprotective properties with efficacy similar to a clinically used drug. A similar approach in the therapy of acute kidney injury was recently adapted by Chen et al. where they applied rectangular DONs (rDONs) by upgrading as a nanodevice with anti-C5a aptamers (aC5a) to block the C5a-mediated inflammation [129]. aC5a-rDONs allowed for the local protection from oxidative stress by scavenging ROS in stage I and suppress the inflammatory responses by blocking C5a in stage II in a renal ischemia-reperfusion (I/R model).

Similar to the application of other DNSs to deliver the small RNA molecules into the cells, the potential application of tFNA has been extended to apply to deliver microRNA (miR). Recently, Li et al. applied tFNA to deliver miR-2861 (model miR) to target the expression of histone deacetylase 5 (HDAC5) in bone marrow mesenchymal stem cells [130]. To achieve the successful separation of the miRs from tFNA after the cell entry, they connected a sticky-end tFNA and miR-2861 via an RNaseH-responsive sequence. They fabricated this bioswitchable delivery system with (i) the protection of miRs in the form of double-stranded RNA, (ii) the transport of miRs with tFNA cell-entrance ability, (iii) RNaseH attribute to unload the miRs to avoid the involvement of tFNA in subsequent biochemical reactions, and (iv) the thermodynamic stability of the 5′ end of the guide strand enables the formation of the RISC. The images of hematoxylin and eosin (H&E) staining showed that the regenerated bone tissue in the stFNA-miR group had filled the entire bone defect area while other groups exhibited some non-osteogenic areas. Masson’s trichrome staining showed that the stFNA-miR group exhibited a significantly higher collagen fiber content than the control and other groups after one and two weeks. Another group, Qin et al. developed microRNA-155-equipped tFNAs (T-155) and determined the effects on choroidal neovascularization (CNV) [131]. They targeted macrophages instead of targeting vascular endothelial growth factor. They reported that T-155 can enter the cell and bind to the target gene to reduce its expression while improving the pathology of CNV by polarizing macrophages to M1 type.

Interestingly, Zhang et al. employed tFNA to deliver antisense oligonucleotides (ASOs) against multiple targets in bacterial cells to inhibit biofilm formation [132]. Extracellular polysaccharides (EPS) and bacteria can cause biofilms to become adherent, toxic, resistant to antibiotics, and ultimately difficult to remove. They selected *Streptococcus mutans* (*S. mutans*) biofilm that is related to the onset of various oral disease targeting *gtfBCD*, *gbpB*, and *fif* genes. They demonstrated that ASOs-tFNAs could penetrate the cell wall of *S. mutans*, targeting multiple genes in the early stages of biofilm formation and improving the inhibitory action using confocal and scanning electron microscope. The biofilms treated with 750 nM ASOs-tFNAs showed a significant reduction in EPS with more porous and spongier structure in comparison with tFNAs and ASOs alone treatments. Another application of tFNA was demonstrated to deliver antimicrobial peptides (AMPs) by Liu et al. [133]. They combined tFNA with an AMP, GL13K, and investigated the effects of resultant complexes against *Escherichia coli* (*E. coli*) that is sensitive to GL13K and *Porphyromonas gingivalis* (*P. gingivalis*) that can degrade GL13K. While AMP-tFNA increased the effects against *E. coli*, the tFNA protected the peptides against *P. gingivalis* serving as a suitable delivery vehicle for AMPs targeting a broad range of diseases. These findings highlighted the versatility of tFNA in combating several defects and diseases. Examples of some of the publications that applied tFNAs alone or with modifications for therapeutic purposes are listed in Table 3. For an in-depth report on the design, fabrication, and applications of tFNA-based multifunctional complexes in drug delivery and biomedical treatment, we direct the readers to the intensive work reported by Zhang et al. [134].

## 6. DNA Nanostructures Interacting with the Cell Membrane

In addition to drug delivery into cells, DNSs have been tested to interact with lipid membranes for synthetic biological purposes, such as cell signaling pathways, cell–cell adhesion, and synthetic DNA nanopores in artificial cell systems. It has long been known that cationic lipids can be used to transfect DNA into hard-to-transfect cell types [142] and to deliver siRNA into cells [143,144,145,146] while negatively charged lipids can repel DNA. The affinity between DNA and negatively charged lipids can be enhanced with positively charged divalent cations (Mg^2+^, Ca^2+^) and reduced with monovalent ones (Na^+^, K^+^). While the mechanism is not fully understood, this effect can probably result because divalent cations bridge from the phosphate backbone of DNA to the negatively charged pole of lipid heads. On the other hand, monovalent cations can reduce this affinity with the lack of bridging and the presence of competitive binding. Different lipid states, such as liquid-disordered (Ld) and solid-ordered (So) states, may also influence how DNA origami behaves on the lipid membrane. A demonstration of the lipid phase-dependent behavior of DNA origami structures was achieved using giant unilamellar vesicles (GUVs) and supported lipid bilayers, suggesting that 2D lattices from cross-shaped DNA origami were formed in the Ld phase while DNA origami aggregated in the So phase [147]. In nature, hydrophilic DNA does not interact with or cannot be inserted into the hydrophobic lipid bilayer. Hydrophobic anchor molecules, such as cholesterol, porphyrin, or polypropylene oxide, are required to strengthen the association between DNA structures and lipid membranes.

**Figure 9:**
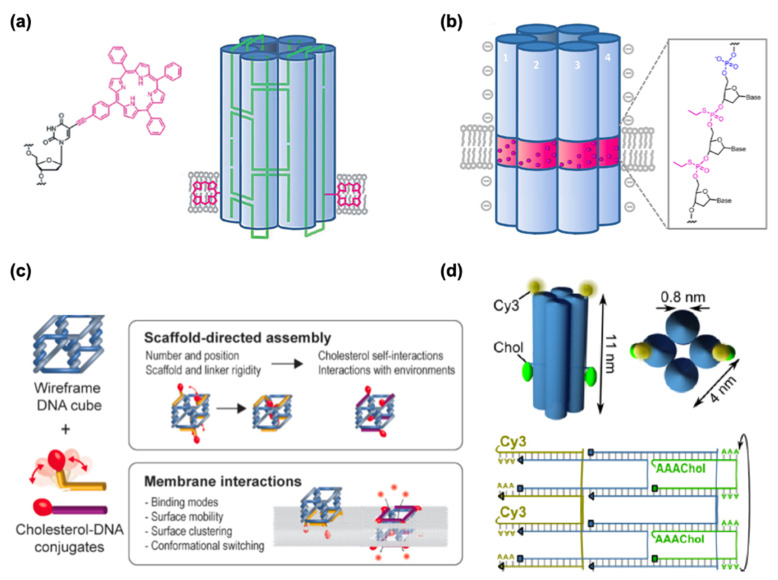
DNA nanostructures interact with the cell membrane. (**a**) DNA-nanopore carrying porphyrin-based lipid anchors. Deoxyuridine bonded to tetraphenylporphyrin (TPP) through an acetylene linkage at the 5 position of nucleobase (Left) A DNA nanopore composed of six interconnected duplexes, drawn as cylinders. (Green—DNA oligonucleotides, Magenta—Porphyrin tags anchoring the DNA nanopore into the lipid bilayer.) Adapted with permission from [148]. Copyright © 2022, The Authors published by Wiley-VCH Verlag GmnH & Co. KGaA. (**b**) A DNA nanopore composed of six interconnected duplexes represented as cylinders with an external face featuring a membrane-spanning hydrophobic belt (magenta) where the conventional phosphate of the DNA backbone is substituted with charge-neutral phosphorothioate-ethyl groups. Adapted with permission from [153]. Copyright © 2022, American Chemical Society. (**c**) A DNA cube with cholesterol anchors in lipid membrane mimicking membrane protein. Adapted with permission from [150]. Copyright © 2022, American Chemical Society. (**d**) Schematic side view (top left) and top view (top right) of the DNA-tile structure composed of four interconnected duplexes represented as cylinders. (Green—cholesterol anchors, Yellow—Cy3-tags) and pathways of eight tiles forming the four duplexes and positions of the Cy3 and cholesterol modifications (Bottom) Adapted with permission from [149]. Copyright © 2022, American Chemical Society.

Cholesterol is the most commonly used membrane anchor because it can easily be attached to DNA at the 5′ or 3′ end during DNA synthesis through a triethyleneglycol spacer. Most DNA nanopores (Table 4) employ various amounts of cholesterol anchors for membrane channels. Burns et al. applied a different approach from cholesterol with porphyrin-based hydrophobic tags to achieve the anchoring of the negatively charge DNA nanopore into the lipid bilayer [148] (Figure 9a). Modifying DNA by altering its chemical properties has also been shown to achieve membrane–DNA interactions, where the hydrophobicity of the DNA was achieved via alkylation (Figure 9b). One of the most common features of DNA nanopore is to allow the ion conduction through lipid bilayers and showing the gating and voltage-switching behavior. Gopfrich et al. demonstrated such function by employing DNA-based membrane channel with openings that are much smaller than a six-helix bundle (Figure 9d) [149]. Chidchob et al. showcased the flexibility to the programmable design featuring a cubic DNA scaffold with cholesterol anchors to act as a mimicking membrane protein with multiple functions (Figure 9c) [150].

One possible application of DNA nanopores in the biomedical field could be to induce cytotoxicity or transport materials through nanopores, such as nucleic acid therapeutics, into the target cells. While most of these DNSs in lipid membranes are designed within synthetic liposomes, the actual cell membrane possesses much more complicated chemical and physical properties than artificial lipid bilayers. Therefore, research on DNA–lipid interactions should also focus on designing nanostructures that interact with native or exogenous cell surfaces to stimulate the cell for intracellular responses and interfere with cellular function. This can also facilitate the delivery of cargos with limited modes of delivery, such as proteins.

Although viral-based vectors can deliver a DNA plasmid that encodes a protein of interest, there can be some adverse side effects, the direct delivery of proteins to modulate cell functions is more straightforward. For example, in the delivery of CRISPR-Cas9, a very effective tool in genome editing, plasmids expressing Cas9 can suffer from a high frequency of off-target effects. The delivery of functional Cas9 has been shown to increase genome modification and specificity compared to DNA transfection [151]. In addition to delivering proteins using lipid nanoparticles, Sun et al. reported a DNA-programmed membrane fusion strategy to deliver proteins into live cells [152]. They employed two single-stranded (ss) DNAs (28 nt) with cholesterol anchors, one at the 3′ cholesterol-functionalized ssDNA (anchor 1) and its complementary 5′ cholesterol-functionalized ssDNA (anchor 2) to mediate fusion between live cell membranes and artificial liposomes (with a mean diameter of 100 nm, for the composition of the lipids). They demonstrated the delivery of cytochrome C into HeLa and L1210 cells and observed a dramatic decrease in cell viability. Their method bypassed the endosome–lysosome–lysosomal escape pathway with a shorter incubation time of 30 min, suggesting a relatively rapid delivery of protein drugs for therapeutic applications.

## 7. Conclusions

In this review, we discussed the potential applications of DNSs for biomedicine and therapeutic purposes. DNSs have become a favorable alternative to other drug carriers owing to their biocompatibility, programmability, and biodegradability. While major publications in the field have focused on cancer therapy as a drug or gene carrier for chemotherapy and gene therapy, the use of DNSs has also been explored in the treatment of other diseases such as Alzheimer’s disease, Parkinson’s disease, and acute kidney disease. Despite the various designs and modifications to DNSs as a drug carrier, most of them have been linked to anti-cancer drugs and ligands that can target molecular markers overexpressed on the surface of cancer cells. However, with the advantage of programmability, DNSs can also be applied as vaccine-carrying materials. DNA structures have been found to effectively circumvent drug resistance in several cells. Because of its programmability, a DNA structure can perform multiple tasks as a single structure by executing multiple therapeutic effects and delivering multiple drugs simultaneously. DNA nanorobots can also be programmed with logic-gated molecular designs to achieve the desired output from single, binary, or multiple inputs.

A major concern for the application of DNSs in biomedical applications is the DNA itself. Even though DNA in nature is hereditably biocompatible and may not result in toxicity in the host compared to other nanomaterials, the actual pharmacokinetics of DNSs in the physical body remains to be elucidated. While DNA itself is biodegradable, its properties can change when it self-assembles into DNSs; hence, systemic studies of the behavior of DNSs in the human body should be performed before they can be commercially formulated as therapeutic drugs. Moreover, self-assembled DNSs are designed to assemble in the presence of a high concentration of divalent cations (such as Mg^2+^) which is incompatible with physiological conditions. When attempting to avoid using Mg^2+^ with monovalent cations such as Na^+^ or K^+^, which are more commonly present in the body, very high concentrations of such monovalent cations are required to achieve a similar effect and doing so can be counterproductive. Decreasing the concentration of divalent cations close to physiological levels can be deleterious to the stability of DNS before they reach their target cells. Moreover, DNSs are mostly assembled via simple base pairing, and one breakage of such linkages can contribute to the gradual destruction of the entire structure.

Another factor is the vulnerability of DNA to nuclease digestion. Nucleases are abundant in the human body [161] and DNSs will inevitably encounter such enzymes during drug delivery. More structurally compacted DNA origami are generally more resistant to enzyme degradation than linear DNA strands because it takes longer to digest larger DNSs than regular DNA strands. In addition to enzymes, DNS encounters the immune system, which recognizes such structures as foreign materials. To maintain structural integrity and avoid immune recognition, DNSs can be encapsulated in a lipid bilayer to mimic the morphology of viruses. While such modifications are applicable, they will still undermine the ability of DNSs to become a clinical therapeutic agent. In contrast, the design of hybrid systems between DNA structures and other drug vehicles such as polymers, liposomes, and viruses, can focus on the overall efficiency improvement of drug carriers.

Most studies have shown that cells take up DNSs through a limited endocytic pathway. Inside the cell, how DNSs escape endosomes and how much ends up in lysosomes is still unclear. Consequently, the amount of DNS required for a payload to deliver an efficient and adequate effect is unknown, leading to the potential overloading of the drug or payload. To be approved as a clinical drug, in the case of liposomes, the weight-to-weight ratio of drug and lipid should be over 70% to avoid high lipid concentration in the circulation [162]. Considering this, DNSs of a simple design with less structural complexity and lower molecular weight are more desirable for clinical formulations to reduce the saturation of DNSs in the circulation and minimize unspecific effects. The folding of most origami DNSs depends heavily on the limited number of scaffold species. To address this issue, researchers have focused on developing more economical approaches for the synthesis of scaffolds, such as the application of a polymerase chain reaction [163], rolling circle amplification [164], and the mass production of bacteriophage-derived scaffold molecules [165]. Another concern is the tendency of intercalating drugs to self-associate in aqueous solutions. Drug escape from DNSs could lead to an early release in the circulation, limiting the controlled release of the payload, which is another important factor for using nanocarriers.

DNA nanotechnology is a relatively new field that will inevitably face obstacles and challenges in adapting to practical applications. However, DNSs have prominent features and advantages, such as programmability to carry multiple drugs or multiple types of therapeutics, relatively less toxicity, biocompatibility, and the ability to act as a smart therapeutic or intelligent nanorobot. Over the recent decades, DNSs have shown improvements from in vitro to in vivo applications. DNA structures of various sizes and shapes have been tested to carry several payloads, including small-molecule drugs, aptamers, CpG sequences, and antibodies. Several studies have proven that MDR can be overcome by delivering small-molecule drugs loaded in DNSs. More findings suggest an improvement in the specificity and cellular uptake of the payload distributed by DNA nanocarriers. Coupled therapy with multiple payloads or combined therapeutic effects and pathways can result in an overall improved efficiency of fighting against diseases. Several modifications, such as coating DNA origami with proteins, viral capsids, lipids, and polymers, have been made to avoid the adverse effects of nuclease digestion and immune response, as well as to maintain structural integrity. Therefore, with the immense research and current trends in DNA nanotechnology, after the fundamental issues have been addressed, DNA nanocarriers show promise in useful applications for biomedical and biomolecular engineering.

## Figures and Tables

**Table 1:** Strategies to modulate nuclease resistance in a living cell.

Structure	Size (nm)	Strategy	Test	Results		Ref.
				Before Modification	After Modification	
24-HB	100	Close-packed helices	DNase I	Duplex plasmid DNA degraded in 5 min	Close-pack helices degraded in 1 h	[36]
Tweezers	14	Topology	70% human serum	Open state in 20 h	Closed state in 37 h	[37]
Paranemic crossover (PX), Double crossover (DX), Duplex DNA	13	Increased crossovers	10% FBS, human serum and urine, DNase I, Exonuclease V, T5 and T 7		PX–not degraded DX–not degraded Duplex–degraded	[38]
Octahedron	50	Heating FBS	Media + 10% FBS	0% intact without heating	100% intact with heating	[33]
Octahedron	50	Nuclease inhibitors	Media + 10% FBS	0% intact without acting	100% intact with actin	[33]
Nanotube	400					
Nanorod	89					
Tetrahedron	14	Ethylenediamine buffer	DNase I	0% intact in TAE with Mg^2+^ buffer	100% intact in ethylenediamine buffer	[39]
Nanotube	30	Crosslinking (Click chemistry)	Exonuclease	Fully degraded	Partially degraded for crosslinked	[40]
Brick-like DNA origami	70	Crosslinking (UV-induced T-T dimers)	DNase I	10 min	1 h	[41,42]
Triangular prism, tetrahedron	7	Hexanediol and hexamethylene glycol	Media + 10% FBS	18 h lifetime	55 h lifetime	[43,44]
DNA brick	50	Dendritic oligonucleotides	DNase I (100 U/mL)	Fully degraded with 5 U/mL	Coated— 50% degraded with 100 U/mL	[45]
Origami rod	350	Cationic polysaccharides	DNase I	Stable for 1 h	Stable for 24 h	[46]
Origami barrel	60	Oligolysine-PEG copolymer	Media + 10% FBS	5 min half-life	50 min half-life	[47]
Octahedron	76	PEGylated lipid bilayer	DNase I	30% intact	85% intact	[29]
60 HB	20×	BSA-dendron conjugates	Media + 10% FBS	20% intact	100% intact	[31]
	20×					
	33					
24 HB	100	Silica coating	DNase I	Completely degraded	Almost fully intact	[48]
Octahedron	29	Peptides	DNase I	Completely degraded	Almost fully intact	[48]
4-Arm junction Nanotube	5 30–70	L-DNA (mirror form of D-DNA)	Exonuclease I Exonuclease III	Completely degraded	Almost fully intact	[42]

**Table 3:** Tetrahedral framework nucleic acids applied as therapeutic agents in neural diseases.

tFNA Design	Targeted Disease	Results	Ref.
tFNA with aptamer conjugation	Cerebral ischemia-reperfusion	Alleviate oxidative stress	[116]
tFNA-aptamer to deliver siRNA	Glioma cells	Apoptosis	[117]
tFNA	Alzheimer’s disease	Apoptosis	[135]
tFNA with aptamer and paclitaxel nanoconjugates	Glioblastoma	Apoptosis	[120]
tFNA loaded with Temozolomide	Glioblastoma	Apoptosis, Autophagy	[70]
tFNA	Parkison’s disease	Apoptosis, differentiation	[135]
tFNA	Alzheimer’s disease	Apoptosis	[136]
tFNA	Retinal ischemia-reperfusion	Apoptosis	[137]
tFNA	Spinal cord injury	Apoptosis	[138]
tFNA loaded with SiCCR2	Intracranial hemorrhage	Anti-inflammation	[139]
tFNA	Facial nerve injury	Proliferation, differentiation	[124]
tFNA with microRNA-22-3p	Glaucoma	Apoptosis, proliferation	[140]
tFNA with Vitamin B12	Parkinson’s disease	Autophagy, proliferation, differentiation	[141]

**Table 4:** Designs and structures of DNA nanopores.

DNA Nanopore Design	Membrane Anchor	a*	b*	c*	Notable Feature	Ref.
Four-helix bundle	Cholesterol	0.8	11	4	Ion conduction through a lipid bilayer	[149]
Six-helix bundle	Cholesterol	2	9	3	Selective transport of small molecules with different charge	[154]
Barrel shape	Cholesterol	2	47	26	Transport of DNA hairpin and G-quadruplex	[155]
Square funnel shape	Cholesterol	6 × 6	54	19	The first largest synthetic pore	[156]
Wireframe cube	Cholesterol	7 × 7	7	8	First open-walled DNA nanopore	[150]
Single duplex	Tetraphenylporphyrin		5	6	Ion-channel made from single DNA duplex	[157]
Six-helix bundle	Tetraphenylporphyrin	2	14	2	Nanopore with two bifunctional tags	[148]
Six-helix bundle	Tetraphenylporphyrin	2	14	2	Low conductance occurs at a higher voltage	[158]
Six-helix bundle	Alkylphosphorothiolates	2	15	72	Nanopore with modified DNA hydrophobicity	[153]
Six-helix bundle 4 × 4 double helix octagon	Alkylphosphorothiolates Cholesterol	2 35	15 10	72 32	Design Simulation Transport of large macromolecules such as folded proteins	[159] [160]

a* Pore size (inner diameter or width by design) in nm, b* channel length (including transmembrane and extra-membrane domains) in nm, c* Number of anchors.
